# Mode of Death Among Japanese Adults With Heart Failure With Preserved, Midrange, and Reduced Ejection Fraction

**DOI:** 10.1001/jamanetworkopen.2020.4296

**Published:** 2020-05-07

**Authors:** Takeshi Kitai, Chisato Miyakoshi, Takeshi Morimoto, Hidenori Yaku, Ryosuke Murai, Shuichiro Kaji, Yutaka Furukawa, Yasutaka Inuzuka, Kazuya Nagao, Yodo Tamaki, Erika Yamamoto, Neiko Ozasa, W. H. Wilson Tang, Takao Kato, Takeshi Kimura

**Affiliations:** 1 Department of Cardiovascular Medicine, Kobe City Medical Center General Hospital, Kobe, Japan; 2 Center for Clinical Research and Innovation, Kobe City Medical Center General Hospital, Kobe, Japan; 3 Department of Clinical Epidemiology, Hyogo College of Medicine, Nishinomiya, Japan; 4 Department of Cardiovascular Medicine, Kyoto University Graduate School of Medicine, Kyoto, Japan; 5 Department of Cardiology, Kurashiki Central Hospital, Kurashiki, Japan; 6 Department of Cardiology, Shiga General Hospital, Moriyama, Japan; 7 Division of Cardiology, Osaka Red Cross Hospital, Osaka, Japan; 8 Division of Cardiology, Tenri Hospital, Tenri, Japan; 9 Kaufman Center for Heart Failure, Department of Cardiovascular Medicine, Heart and Vascular Institute, Cleveland Clinic, Cleveland, Ohio

## Abstract

**Importance:**

Despite intensive treatment, hospitalized patients with acute decompensated heart failure (ADHF) have a substantial risk of postdischarge mortality. Limited data are available on the possible differences in the incidence and mechanisms of death among patients with heart failure with reduced ejection fraction (HFrEF), heart failure with midrange ejection fraction (HFmrEF), and heart failure with preserved ejection fraction (HFpEF).

**Objectives:**

To examine the incidences and mode of postdischarge mortality among patients with ADHF and to compare the risk profile among patients with HFrEF, HFmrEF, and HFpEF.

**Design, Setting, and Participants:**

This prospective cohort study of 4056 patients hospitalized for ADHF analyzed data from 3717 patients who were discharged from October 1, 2014, to March 31, 2016. Data analysis was performed from April 1 to August 31, 2019.

**Exposures:**

Death among patients with ADHF after hospital discharge.

**Main Outcomes and Measures:**

All-cause death and cause of postdischarge mortality after the index hospitalization by left ventricular ejection fraction (LVEF) subgroup.

**Results:**

A total of 3717 patients (mean [SD] age, 77.7 [12.0] years; 2049 [55.1%] male) were included in the study. The mean (SD) LVEF at baseline was 46.4% (16.2%). Among 3717 enrolled patients, 1383 (37.2%) were categorized as having HFrEF (LVEF, <40%), 703 (18.9%) as having HFmrEF (LVEF, 40%-49%), and 1631 (43.9%) as having HFpEF (LVEF, ≥50%). The incidence and causes of death were evaluated after discharge from the index hospitalization. The median follow-up period was 470 days (interquartile range, 357-649 days), and the 1-year follow-up rate was 96%. During follow-up, all-cause death occurred in 848 patients (22.8%; HFrEF group: 298 [21.5%; 95% CI, 19.5%-23.8%]; HFmrEF group: 158 [22.5%; 95% CI, 19.5%-25.7%]; and HRpEF group: 392 [24.0%; 95% CI, 22.0%-26.2%]; *P* = .26), cardiovascular deaths occurred in 523 patients (14.1%; HFrEF group: 203 [14.7%; 95% CI, 12.9%-16.6%]; HFmrEF group: 97 [13.8%; 95% CI, 11.4%-16.5%]; and HFpEF group: 223 [13.7%; 95% CI, 12.1%-15.4%]; *P* = .71), and sudden cardiac death occurred in 98 patients (2.6%; HFrEF group: 44 [3.2%; 95% CI, 2.4%-4.2%]; HFmrEF group: 14 [2.0%; 95% CI, 1.2%-3.3%]; and HFpEF group: 40 [2.5%; 95% CI, 1.8%-3.3%]; *P* = .23). The risks of causes of death were similar among the subtypes.

**Conclusions and Relevance:**

The mode of death was similar among the heart failure subtypes. Given the nonnegligible incidence of sudden cardiac death in patients with HFpEF found in this study, further studies appear to be warranted to identify a high-risk subset in this population.

## Key Points

**Question:**

Are there differences in the mode of death after hospital discharge in patients with reduced, midrange, and preserved left ventricular ejection fraction?

**Findings:**

In this cohort study of 3717 hospitalized patients with acute decompensated heart failure with a median follow-up of 470 days, 848 patients died (523 cardiovascular deaths and 98 sudden cardiac deaths). The risks of each cause of death were comparable among the patients with heart failure with reduced, midrange, and preserved ejection fraction.

**Meaning:**

This study found nonnegligible incidence of sudden cardiac death in patients with heart failure with preserved ejection fraction; further study appears to be warranted to identify a high-risk subset in this population.

## Teaser

This cohort study of Japanese patients with acute decompensated heart failure examines the incidence and mode of postdischarge mortality among patients with reduced, midrange, and preserved ejection fraction.

## Introduction

Heart failure has been an increasing public health concern, and hospitalization rates and costs of care for heart failure remain high.^1^ Substantial progress has been made in the management of chronic ambulatory heart failure with the availability of drugs such as β-blockers, angiotensin-converting enzyme inhibitors (ACEIs), angiotensin receptor blockers (ARBs), and mineralocorticoid receptor antagonists (MRAs). However, morbidity and mortality among patients with heart failure are still high.^2,3,4,5^ Hospitalized patients with acute decompensated heart failure (ADHF) had an annual mortality rate of approximately 20%, which is higher than the rates among patients with chronic ambulatory heart failure.^6,7^ However, the incidence and mechanisms of death among patients with ADHF who are discharged from the hospital have not been well characterized. A better understanding of the cause and mode of death in these patients may lead to better insights into the underlying pathophysiologic mechanisms and new treatments for improving patient outcomes. In addition, limited data are available for the possible differences in the mode of mortality among patients with heart failure with reduced ejection fraction (HFrEF), heart failure with midrange ejection fraction (HFmrEF), and heart failure with preserved ejection fraction (HFpEF). Therefore, we aimed to assess the prevalence and mode of mortality among patients with ADHF hospital after discharge and then compare the risk profile among patients with HFrEF, HFmrEF, and HFpEF.

## Methods

### Study Design

The study design and primary results of the Kyoto Congestive Heart Failure (KCHF) registry have been reported previously.^8,9^ In brief, the KCHF registry was a multicenter, prospective cohort study that enrolled 4056 consecutive hospitalized patients with ADHF. The study was conducted from October 1, 2014, to March 31, 2016, at 19 centers in Japan after approval of each participating center’s ethics committee or institutional review board. A waiver of informed consent was granted by the institutional review boards because the study met the conditions of the Japanese ethical guidelines for epidemiologic study and the US policy for protecting human research participants. This prespecified post hoc analysis was approved by institutional review boards of each participating institution. This study followed the Strengthening the Reporting of Observational Studies in Epidemiology (STROBE) reporting guideline.

Among the 4056 enrolled patients in the KCHF registry, 3785 patients (93.3%) were discharged after the index hospitalization for ADHF. Clinical follow-up data were collected in October 2017, and the median follow-up period was 470 days. The attending physicians or research assistants at each participating facility collected clinical events data after the index hospitalization from hospital medical records or from patients, their relatives, or their referring physicians (with patient consent).

After excluding 57 patients who were unavailable for follow-up after discharge and 11 patients who had a missing left ventricular ejection fraction (LVEF) measurement at baseline, a total of 3717 patients were included in the current analysis. Patients were divided based on their LVEF at baseline: less than 40% (HFrEF), 40% to 49% (HFmrEF), and 50% or higher (HFpEF). The eFigure in the Supplement shows the selection of these patients from the overall KCHF population. Data analysis was performed from April 1 to August 31, 2019.

Patients’ baseline characteristics, including age, height, body weight, blood pressure, heart rate, laboratory data, and echocardiographic data, were recorded or measured at the time of hospital discharge. A baseline medication was defined as a medication at the time of discharge. Incident death and the cause of death were adjudicated up to 1 year. The causes of death were adjudicated by a central clinical events committee on the basis of prespecified criteria and were classified into cardiovascular death or noncardiovascular death. Cardiovascular death comprised death due to heart failure exacerbation, acute coronary syndrome, stroke and intracranial hemorrhage, or fatal ventricular arrhythmia; vascular-related death; sudden cardiac death (SCD); and other cardiac death causes. SCD was defined as unexplained death of a previously stable patient, including fatal ventricular arrhythmia and cardiac arrest. Noncardiovascular deaths included malignant tumors, infection (including pneumonia), renal failure, liver failure, respiratory failure, bleeding, and other causes.

### Statistical Analysis

Categorical variables are expressed as numbers (percentages) and were compared using the χ^2^ test or the Fisher exact test, as appropriate. Continuous variables are expressed as means (SDs) or medians and interquartile ranges. On the basis of their distribution (qualitatively judged by histogram and Q-Q plot), continuous variables were compared with an unpaired, 2-tailed *t* test when normally distributed or with the Wilcoxon rank sum test when not normally distributed. Two-sided *P* < .05 was considered statistically significant. The Kaplan-Meier method was used to estimate cumulative incidence of events, and differences were compared using the log-rank test. A Cox proportional hazards regression model was used to evaluate the association between each variable and the incidence of all-cause death, cardiovascular death, and noncardiovascular death. Candidate variables for the multivariable model included age, sex, hypertension, diabetes, atrial fibrillation, anemia, chronic kidney disease, serum albumin level, blood urea nitrogen (BUN) level, and prescription of β-blockers, ACEIs or ARBs, and MRAs at discharge. All variables were selected a priori because they are risk factors for death or because of their ability to confound the association. Proportional hazards assumption violations were estimated by generalized linear regression of scaled Schoenfeld residuals on time. Continuous variables were dichotomized by median values or clinically meaningful reference values.

We introduced a bayesian network to estimate associations between risk factors and mortality. A bayesian network is a probabilistic graphical model in which conditional dependencies among multiple factors are represented by edges. We constructed a bayesian network and assumed multinomial distribution for the outcome variable and binomial distribution for the other variables. With the use of the data without any missing values, the posterior distributions of variables were obtained using the Markov chain Monte Carlo method. We set 4 separate sampling sequences, each consisting of 1000 random samples, half of which were discarded for convergence. Sampling convergence was evaluated using Gelman-Rubin statistics and by visually inspecting trace plots. All prior variables were set as noninformative.

Statistical analyses were performed using JMP, version 14.0.0 (SAS Institute Inc) and R, version 3.5.1 (R Foundation for Statistical Computing), with probabilistic programming language Stan (Stan Development Team) for all bayesian analyses.

## Results

### Patient Characteristics

A total of 3717 patients (mean [SD] age, 77.7 [12.0] years; 2049 [55.1%] male) were included in the study. A total of 1000 patients (26.9%) had ischemic heart failure. The mean (SD) heart rate was 71/min (13/min), the mean (SD) systolic blood pressure was 116 (18) mm Hg, and the mean (SD) diastolic blood pressure was 64 (12) mm Hg. The mean (SD) LVEF at baseline was 46.4% (16.2%). Among the 3717 enrolled patients, 1383 (37.2%) were categorized as having HFrEF (LVEF, <40%), 703 (18.9%) as having HFmrEF (LVEF, 40%-49%), and 1631 (43.9%) having as having HFpEF (LVEF, ≥50%).

Comparisons of baseline patient characteristics among the 3 groups and missing values in each variable are given in Table 1. Older age was associated with increased likelihood of LVEF (mean [SD] age in HFrEF group: 73.8 [13.6] years; mean [SD] age in HFmrEF group: 78.1 [11.0] years; and mean [SD] age in HFpEF group: 80.7 [9.9] years; *P* < .001), and an increased prevalence of LVEF among women was observed (HFrEF group: 458 [33.1%]; HFmrEF: 283 [40.3%]; and HFpEF: 927 [56.8%]; *P* < .001). An ischemic origin was most frequent in patients with HFrEF, whereas hypertension and atrial fibrillation were most frequent in patients with HFpEF.

**Table 1.** Baseline Characteristics and Medications at Discharge^a^

Characteristic	All (N = 3717)	HFrEF group (n = 1383)	HFmrEF group (n = 703)	HFpEF group (n = 1631)	*P* value
Age, mean (SD), y	77.7 (12.0)	73.8 (13.6)	78.1 (11.0)	80.7 (9.9)	<.001
Male	2049 (55.1)	925 (66.9)	420 (59.7)	704 (43.2)	<.001
BMI, mean (SD)	22.9 (4.5)	22.9 (4.6)	22.7 (4.2)	23.0 (4.4)	.43
LVEF, mean (SD), %	46.4 (16.2)	29.1 (7.1)	44.3 (2.9)	61.9 (7.5)	<.001
Ischemic origin	1000 (26.9)	534 (38.6)	234 (33.3)	232 (14.2)	<.001
Blood pressure, mean (SD), mm Hg					
Systolic	116 (18)	112 (17)	119.5 (17.9)	118 (18)	<.001
Diastolic	64 (12)	64 (13)	65 (12)	64 (12)	.007
Heart rate, mean (SD), /min	71 (13)	72 (13)	71 (12)	70 (13)	<.001
Comorbidities					
Hypertension	2690 (72.4)	911 (65.9)	536 (76.2)	1243 (76.2)	<.001
Diabetes	1392 (37.4)	567 (41.0)	286 (40.7)	539 (33.0)	<.001
Dyslipidemia	1452 (39.1)	582 (42.1)	293 (41.7)	577 (35.4)	<.001
Atrial fibrillation or flutter	1550 (41.7)	438 (31.7)	292 (41.5)	820 (50.3)	<.001
COPD	304 (8.2)	107 (7.7)	47 (6.7)	150 (9.2)	.1
Malignant tumor	535 (14.4)	180 (13.0)	104 (14.8)	251 (15.4)	.17
Anemia^b^	2546 (68.5)	843 (61.0)	485 (69.0)	1218 (74.7)	<.001
CKD^c^	1637 (44.0)	588 (42.5)	333 (47.4)	716 (43.9)	.11
Laboratory data, median (IQR)					
BNP level, pg/mL	269 (136-522)	369 (194-664)	294 (152-578)	199 (96-384)	<.001
BUN level, mg/dL	25.2 (18.6-36.0)	24.9 (18.4-34.2)	26.0 (18.8-38.4)	26.0 (18.7-36.4)	.15
Creatinine level, mg/dL	1.12 (0.86-1.59)	1.14 (0.87-1.59)	1.17 (0.86-1.72)	1.10 (0.83-1.54)	<.001
eGFR, mL/min/1.73 m^2^	43.3 (29.3-59.0)	45.3 (30.5-61.0)	42.8 (25.9-58.6)	41.3 (29.2-57.0)	.002
Hemoglobin level, g/dL	11.3 (9.9-12.8)	11.8 (10.4-13.6)	11.2 (9.7-12.8)	10.9 (9.6-12.3)	<.001
Sodium level, mEq/L	139 (136-141)	139 (136-141)	139 (136-141)	139 (137-141)	.005
Albumin level, g/dL	3.4 (3.0-3.7)	3.4 (3.1-3.7)	3.4 (3.0-3.7)	3.4 (3.0-3.7)	.003
Medications					
β-Blocker	2469 (66.4)	1080 (78.1)	504 (71.7)	885 (54.3)	<.001
ACEI or ARB	2138 (57.5)	892 (64.5)	400 (56.9)	846 (51.9)	<.001
MRA	1678 (45.1)	722 (52.2)	310 (44.1)	646 (39.6)	<.001

Abbreviations: ACEI, angiotensin-converting enzyme inhibitor; ARB, angiotensin receptor blocker; BMI, body mass index (calculated as weight in kilograms divided by height in meters squared); BNP, brain-type natriuretic peptide; BUN, blood urea nitrogen; CKD, chronic kidney disease; COPD, chronic obstructive pulmonary disease; eGFR, estimated glomerular filtration ratio; HFmrEF, heart failure with midrange ejection fraction; HFpEF, heart failure with preserved ejection fraction; HFrEF, heart failure with reduced ejection fraction; LVEF, left ventricular ejection fraction; MRA, mineralocorticoid receptor antagonist.

SI conversion factors: to convert albumin to grams per liter, multiply by 10; BNP to nanograms per liter, multiply by 1; BUN to millimoles per liter, multiply by 0.357; hemoglobin to grams per liter, multiply by 10; and sodium to millimoles per liter, multiply by 1.

^a^ Data are presented as number (percentage) of patients unless otherwise indicated.

^b^ Defined by the World Health Organization criteria (hemoglobin <12 g/dL for women and <13 g/dL for men).

^c^ Defined as an eGFR less than 60 mL/min/1.73 m^2^.

### Incidence of Death

The median follow-up period was 470 days (interquartile range, 357-649 days) after discharge, and the 1-year follow-up rate was 96%. During the follow-up period, 848 deaths were observed, and the overall mortality rate was 22.8%. Causes of death were adjudicated as cardiovascular deaths in 523 patients (14.1%; 61.7% of total mortality) and noncardiovascular deaths in 322 patients (8.7%; 38.0% of total mortality). The causes of cardiovascular death included heart failure exacerbation in 324 patients (8.7%), SCDs in 98 patients (2.6%), stroke or intracranial hemorrhage in 38 patients (1.0%), acute coronary syndrome in 9 patients (0.2%), and vascular-related deaths in 13 patients (0.3%). The causes of noncardiovascular death included infection in 122 patients (3.3%), malignant tumor in 71 patients (1.9%), and respiratory failure in 30 patients (0.8%) (Table 2).

**Table 2.** Comparisons of Mode of Death Among the 3 Study Groups

Mode of death	Patients, No. (%)				*P* value
	All (N = 3717)	HFrEF group (n = 1383)	HFmrEF group (n = 703)	HFpEF group (n = 1631)	
All-cause death	848 (22.8)	298 (21.5)	158 (22.5)	392 (24.0)	.26
Cardiovascular	523 (14.1)	203 (14.7)	97 (13.8)	223 (13.7)	.71
Heart failure	324 (8.7)	128 (9.3)	65 (9.2)	131 (8.0)	.42
Sudden cardiac	98 (2.6)	44 (3.2)	14 (2.0)	40 (2.5)	.23
Vascular death	13 (0.3)	4 (0.3)	2 (0.3)	7 (0.4)	.77
Acute coronary syndrome	9 (0.2)	5 (0.4)	0 (0.0)	4 (0.2)	.28
Stroke or intracranial hemorrhage	38 (1.0)	8 (0.6)	9 (1.3)	21 (1.3)	.12
Other cardiovascular cause	41 (1.1)	14 (1.0)	7 (1.0)	20 (1.2)	.82
Noncardiovascular cause	322 (8.7)	94 (6.8)	61 (8.7)	167 (10.2)	.004
Malignant tumor	71 (1.9)	24 (1.7)	9 (1.3)	38 (2.3)	.20
Infection	122 (3.3)	33 (2.4)	28 (4.0)	61 (3.7)	.06
Fatal bleeding	7 (0.2)	1 (0.1)	2 (0.3)	4 (0.2)	.45
Other gastrointestinal cause	10 (0.3)	3 (0.2)	1 (0.1)	6 (0.4)	.56
Renal failure	18 (0.5)	5 (0.4)	2 (0.3)	11 (0.7)	.33
Liver failure	6 (0.2)	1 (0.1)	1 (0.1)	4 (0.2)	.49
Respiratory failure	30 (0.8)	9 (0.7)	4 (0.6)	17 (1.0)	.36
Other noncardiovascular cause	58 (1.6)	18 (1.3)	14 (2.0)	26 (1.6)	.48
Unknown	3 (0.1)	1 (0.1)	0 (0.0)	2 (0.1)	.63

Abbreviations: HFmrEF, heart failure with midrange ejection fraction; HFpEF, heart failure with preserved ejection fraction; HFrEF, heart failure with reduced ejection fraction.

**Figure 1.**
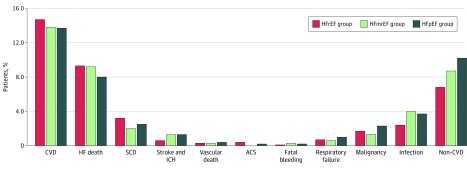
Comparisons of Modes of Death Among Patients in the 3 Study Groups ACS indicates acute coronary syndrome; CVD, cardiovascular death; HFmrEF, heart failure with midrange ejection fraction; and HFpEF, heart failure with preserved ejection fraction; HFrEF, heart failure with reduced ejection fraction; ICH, intracranial hemorrhage; SCD, sudden cardiac death.

**Figure 2.**
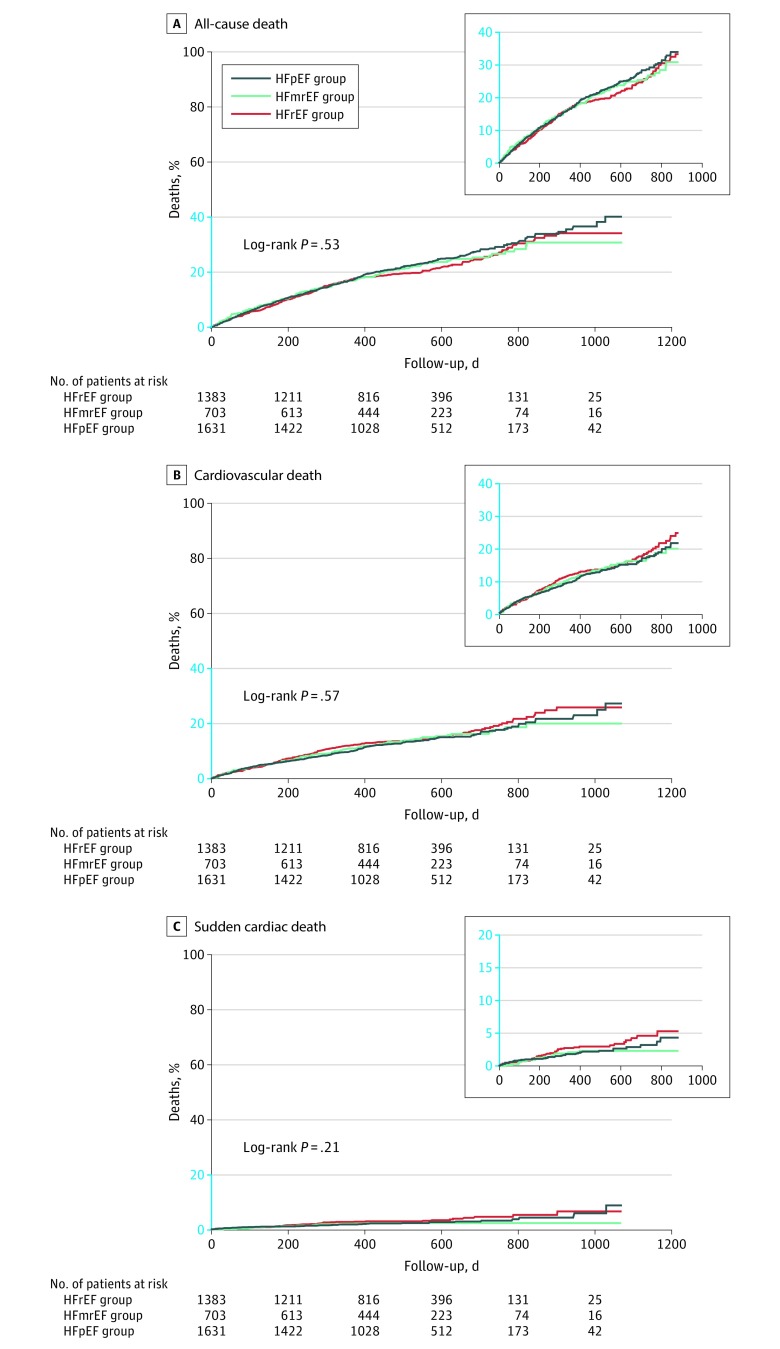
Kaplan-Meier Survival Curves for All-Cause Death, Cardiovascular Death, and Sudden Cardiac Death Among Patients in the 3 Study Groups HFmrEF indicates heart failure with midrange ejection fraction; HFpEF, heart failure with preserved ejection fraction; and HFrEF, heart failure with reduced ejection fraction.

The observed modes of deaths among the 3 groups are compared in Figure 1. No significant differences were found among the 3 groups with respect to all-cause death (HFrEF group: 298 patients [21.6%; 95% CI, 19.5%-23.8%]; HFmrEF group: 158 patients [22.5%; 95% CI, 19.5%-25.7%]; and HFpEF group: 392 patients [24.0%; 95% CI, 22.0%-26.2%]; *P* = .26), cardiovascular death (HFrEF group: 203 patients [14.7%; 95% CI, 12.9%-16.6%]; HFmrEF group: 97 patients [13.8%; 95% CI, 11.4%-16.5%]; and HFpEF group: 223 patients [13.7%; 95% CI, 12.1%-15.4%]; *P* = .71), and SCD (HFrEF group: 44 patients [3.2%; 95% CI, 2.4%-4.2%]; HFmrEF group: 14 patients [2.0%; 95% CI, 1.2%-3.3%]; and HFpEF group: 40 patients [2.5%; 95% CI, 1.8%-3.3%]; *P* = .23). Figure 2 shows the Kaplan-Meier survival curves for all-cause death, cardiovascular death, and noncardiovascular death among the 3 groups.

### Factors Associated With Each Mode of Death

In the multivariable Cox proportional hazards regression analyses, the factors confirmed as the independent variables associated with all-cause death in all the study patients were older age, female sex, no prescription of ACEIs or ARBs, anemia, low albumin levels, high BUN levels, and low estimated glomerular filtration rate (eGFR) (eTable 1 in the Supplement). Among these variables, older age, no prescription of ACEIs or ARBs, low albumin levels, and high BUN levels were consistently associated with all-cause death in all subgroups. Some of these same factors, including older age, no prescription of ACEIs or ARBs, and high BUN levels, were consistently associated with cardiovascular death in the entire population and the subgroups (eTables 2-4 in the Supplement). In addition, factors such as no prescription of β-blockers or MRAs, anemia, and low eGFR were independently associated with cardiovascular death in the HFrEF group. Some of these same factors, including low eGFR and no prescription of MRAs, were independently associated with cardiovascular death in the HFpEF group. Older age, female sex, anemia, low albumin levels, high BUN levels, and no prescription of ACEIs or ARBs were also associated with noncardiovascular death.

**Figure 3.**
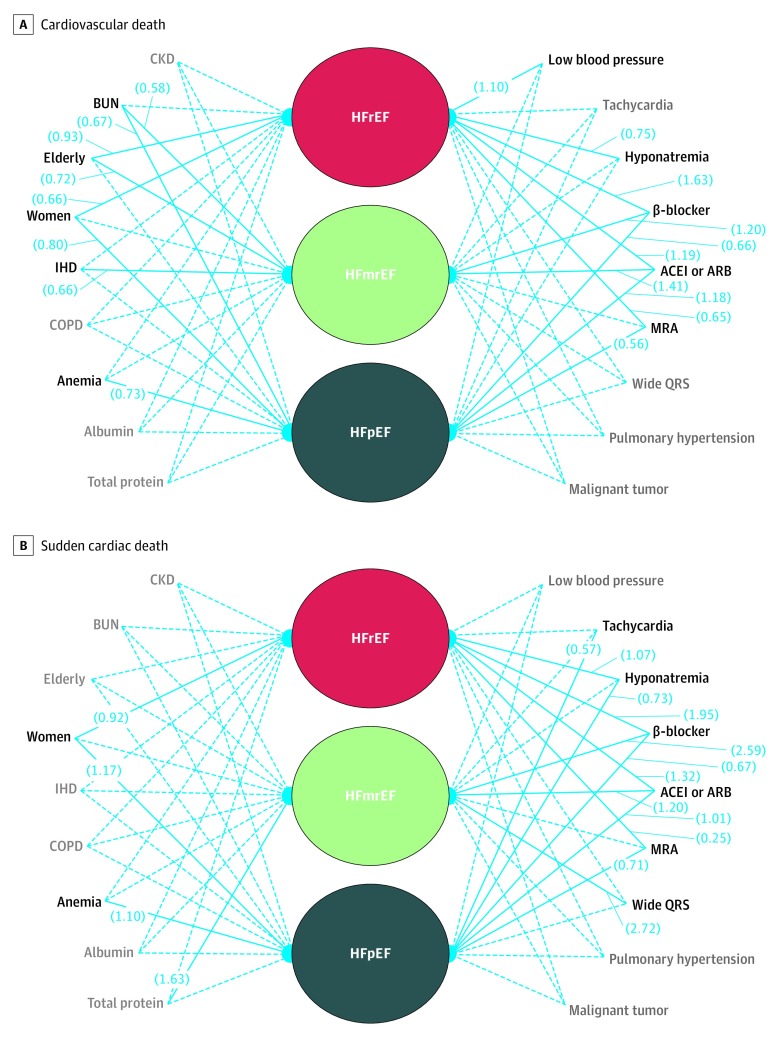
Bayesian Modeling for Cardiovascular Death and Sudden Cardiac Death Among Patients in the 3 Study Groups Solid lines indicate that the association between factors and mortality in each study group are statistically significant, and dashed lines indicate that those associations are not statistically significant. Factors are shown in black if they have at least one significant association with mortality, and factors are shown in gray if they do not have any significant associations with mortality in each study group. Numbers in parentheses indicate path coefficients of each factor to the mortality in each study group. ACEI indicates angiotensin-converting enzyme inhibitor; ARB, angiotensin receptor blocker; BUN, blood urea nitrogen; CKD, chronic kidney disease; COPD, chronic obstructive pulmonary disease; HFmrEF, heart failure with midrange ejection fraction; HFpEF, heart failure with preserved ejection fraction; HFrEF, heart failure with reduced ejection fraction; IHD, ischemic heart disease; MRA, mineralocorticoid receptor antagonist.

The results of bayesian modeling for estimating cardiovascular death and SCD are shown in Figure 3. Guideline-directed heart failure medications, such as β-blockers, ACEIs or ARBs, and MRAs, were associated with a lower incidence of SCD in patients with HFrEF and in patients with HFpEF. Other factors associated with an increased risk of SCD were hyponatremia, HFrEF in female patients, hypoalbuminemia and wide QRS in patients with HFmrEF, increased heart rate, and hyponatremia and female sex in patients with HFpEF. Similarly, β-blockers, ACEIs or ARBs, and MRAs were also associated with a lower incidence of cardiovascular death in the HFrEF group and in the HFpEF group.

## Discussion

The current analysis investigated the postdischarge mode of death in 3717 hospitalized patients with ADHF and among LVEF subgroups (HFrEF, HFmrEF, and HFpEF). The major findings of this study were as follows: (1) overall mortality in hospitalized patients with ADHF after discharge was 22.8% during a median follow-up of 470 days with a 96% follow-up rate; (2) cardiovascular deaths accounted for 61.7% of total mortality and noncardiovascular deaths accounted for 38.0% of total mortality; (3) heart failure exacerbation was the leading cause of cardiovascular death, and SCD was the second most frequent cause of cardiovascular death; and (4) this finding was consistent among the LVEF subgroups (HFrEF vs HFmrEF vs HFpEF), with the risk of SCD being comparable in the HFpEF and HFrEF groups.

ADHF is a complex clinical syndrome, and multiple factors and underlying mechanisms may contribute to postdischarge mortality in individual patients.^10,11,12^ Despite improvement in intensive treatment of acute phases and multidisciplinary approaches to improve postdischarge outcomes, patients hospitalized for ADHF have a substantial mortality risk of 10% to 20% during the 6 months after discharge.^2,3,4,5^ Thus, a better understanding of the mode of death and a better characterization of risks associated with mode-specific causes of death may provide insights into the underlying mechanism to improve patient outcomes. In particular, comparisons of the mode of death among strictly defined populations with HFrEF, HFmrEF, and HFpEF are important for clinical practice.

In the Efficacy of Vasopressin Antagonism in Heart Failure Outcome Study With Tolvaptan (EVEREST) trial, which included 4133 patients with HFrEF hospitalized for ADHF, 1080 deaths occurred during a median follow-up of 9.9 months. Heart failure exacerbation was the leading cause of death (47.2%), and SCD was the second-leading cause of death (30.0%).^13^ In the Efficacy, Safety and Tolerability of Serelaxin When Added to Standard Therapy in Acute Heart Failure (RELAX-AHF) trial, which included 1161 patients with acute heart failure, heart failure exacerbation was a leading cause of cardiovascular death (35%), and SCD was the second-leading cause of cardiovascular death (23%).^14^ Similarly, the current analysis demonstrated that heart failure exacerbation was the leading cause of cardiovascular death, and SCD was the second-leading cause of death. Of interest, SCD was reported to be the second-leading cause of death even in the HFpEF group, and the rate was comparable to that in the HFrEF group. However, the nonnegligible prevalence of SCD is debatable. A similar incidence of SCD was reported in patients with and without left ventricular systolic dysfunction, with a similar potential benefit from implantable cardioverter defibrillator (ICD) prophylaxis.^15^

HFpEF has been reported to be associated with similar or slightly lower mortality than HFrEF.^16,17^ Although heart failure death and SCD account for most cardiovascular deaths among patients with HFpEF, similar to patients with HFrEF,^18^ the major difference in the cause of death between patients with HFrEF and those with HFpEF has been the larger prevalence of noncardiovascular deaths in the HFpEF group.^16,17,19,20^ In the Framingham Heart Study, 1025 deaths in the mixed HFrEF and HFpEF population between 1971 and 2004 were analyzed, and 38% of deaths reportedly had a noncardiovascular mode.^21^ Similarly, another study reported that 40% of deaths were attributable to noncardiovascular modes during 20 months after discharge in 459 patients admitted with ADHF, mixed HFrEF, and HFpEF.^22^ A previous study^23^ reported that 42% of deaths in patients with HFpEF had noncardiovascular causes. Consistent with previous reports,^16,19^ the rate of noncardiovascular death was higher in the HFpEF group in the current analysis. In a previous study,^23^ infection was the leading cause of noncardiovascular death, causing 38% of total noncardiovascular deaths, and malignant tumor was the second-leading cause of noncardiovascular death (22%), findings that were consistent with the those in the LVEF subgroups in the current study.

Reduced LVEF remains the major selection criterion for ICD placement according to the current guidelines,^24^ and increasing evidence supports that ICD is an effective treatment of primary and secondary SCD in patients with left ventricular systolic dysfunction.^25,26^ However, given the substantial amount of SCD observed in patients with LVEFs higher than 35% who do not qualify for ICD placement based on the current criteria,^26,27,28,29^ our results pose the question of whether the ICD criteria should be determined only by LVEF. Although no data are currently available to examine the role of ICD treatment in patients with HFpEF, observational data suggest that SCD contributes substantially to the overall mortality in these patients.^30,31^ However, considering the high incidence of nonarrhythmic heart failure deaths and that ICD placement in patients with HFrEF yielded conflicting results for overall mortality despite increased frequency of adequate ICD shocks, additional studies are needed to identify patients who would optimally benefit from ICD implantation irrespective of the LVEF level.

### Strengths and Limitations

This study has strengths. It provides insight regarding the prevalence, nature, and variables associated with death in patients with postdischarge ADHF, with a high follow-up rate, strictly adjudicated mode of death, and potentially important implications for improvement in survival. The study included central adjudication of end points and a large contemporary patient population across the spectrum of LVEF.

This study has limitations. First, this was a post hoc analysis from a prospective, observational cohort study with inherent associated limitations. Despite covariate adjustment, we could not exclude the influence of other measured and unmeasured confounding. In particular, we did not consider any interim cardiovascular events associated with heart failure death or SCD that may have modified the disease trajectories. Second, it is possible that our data are not generalizable to all patients with ADHF. Particularly, the current cohort included a large number of patients with de novo heart failure rather than acute worsening of chronic heart failure, leading to a small number of patients with ICD implantation at the time of discharge. In addition, the patient population was elderly, and the prevalence of an ischemic origin of heart failure was lower than that reported in other clinical series outside Japan. The diagnosis of nonischemic cardiomyopathy was made by physicians in each participating center, and not all patients with a nonischemic origin underwent coronary angiography during the hospitalization. We did not have data on the number of patients who had ICD implantation during the follow-up after discharge. Third, diagnosis of heart failure origin was not based on biopsy results or imaging findings. In addition, we did not have information regarding whether any of the patients with HFpEF or HFmrEF recovered from HFrEF. In addition, information regarding circumstances of SCD was not available. Thus, there is a possibility that specific patients with cardiomyopathy, such as hypertrophic cardiomyopathy, restrictive cardiomyopathy, and cardiac amyloidosis, were included in the registry. In particular, underdiagnosed cardiac amyloidosis may be associated with a high incidence of SCD in the HFpEF cohort. Additional studies are needed to test this hypothesis.

## Conclusions

In this study, the incidences of cardiovascular death and sudden cardiac death were comparable among the heart failure subtypes. Use of β-blockers and ACEIs or ARBs was associated with lower mortality in patients with HFpEF and HFmrEF. Given the nonnegligible incidence of SCD in patients with HFpEF, an additional study appears to be warranted to identify the high-risk subset in this population.

## Notes

Supplement. **eFigure.** Patient Flowchart **eTable 1.** Multivariable Analyses for Predicting Each Mode of Death in Whole Study Patients **eTable 2.** Multivariable Analyses for Predicting Each Mode of Death in Patients With HFrEF **eTable 3.** Multivariable Analyses for Predicting Each Mode of Death in Patients With HFmrEF **eTable 4.** Multivariable Analyses for Predicting Each Mode of Death in Patients With HFpEF
