# Supplementary material for: Mode of Death Among Japanese Adults With Heart Failure With Preserved, Midrange, and Reduced Ejection Fraction
Source: JAMA Netw Open. 2020 May 7;3(5):e204296. doi: 10.1001/jamanetworkopen.2020.4296 (PMC7206504; doi:10.1001/jamanetworkopen.2020.4296)
Supplement: Supplement. — eFigure. Patient Flowchart eTable 1. Multivariable Analyses for Predicting Each Mode of Death in Whole Study Patients eTable 2. Multivariable Analyses for Predicting Each Mode of Death in Patients With HFrEF eTable 3. Multivariable Analyses for Predicting Each Mode of Death in Patients With HFmrEF eTable 4. Multivariable Analyses for Predicting Each Mode of Death in Patients With HFpEF [file jamanetwopen-3-e204296-s001.pdf]

# Supplementary Online Content

Kitai T, Miyakoshi C, Morimoto T, et al. Mode of death among Japanese adults with heart failure with preserved, midrange, and reduced ejection fraction. *JAMA Netw Open*. 2020;3(5):e204296. doi:10.1001/jamanetworkopen.2020.4296

**eFigure.** Patient Flow Chart

**eTable 1.** Multivariable Analyses for Predicting Each Mode of Death in Whole Study Patients

**eTable 2.** Multivariable Analyses for Predicting Each Mode of Death in Patients With HFrEF

**eTable 3.** Multivariable Analyses for Predicting Each Mode of Death in Patients With HFmrEF

**eTable 4.** Multivariable Analyses for Predicting Each Mode of Death in Patients With HFpEF

**This supplementary material has been provided by the authors to give readers additional information about their work.**

© 2020 Kitai T et al. *JAMA Network Open.*

**eFigure.** Patient Flow Chart

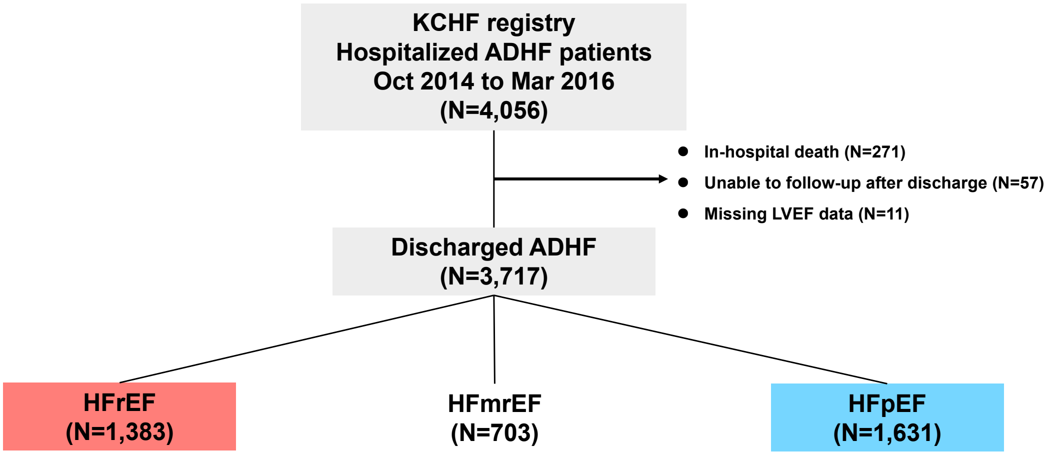


KCHF = Kyoto Congestive Heart Failure; ADHF = acute decompensated heart failure; HFrEF = heart failure with reduced ejection fraction: HFmrEF = heart failure with mid-range ejection fraction; and HFpEF = heart failure with preserved ejection fraction.

© 2020 Kitai T et al. *JAMA Network Open.*

**eTable 1.** Multivariable Analyses for Predicting Each Mode of Death in Whole Study Patients

| | adjusted HR (95%CI) | P value |
|---|---|---|
| **All-cause death** | | |
| Age* | 2.40 (2.01-2.87) | <0.001 |
| Men | 0.80 (0.69-0.93) | 0.001 |
| ACEI/ARB | 0.61 (0.52-0.71) | <0.001 |
| Anemia † | 1.72 (1.39-2.15) | <0.001 |
| Albumin ‡ | 1.77 (1.51-2.07) | <0.001 |
| BUN § | 1.61 (1.35-1.92) | <0.001 |
| eGFR ‖ | 1.26 (1.06-1.51) | 0.017 |
| LVEF category | | |
| HFrEF | reference | |
| HFmrEF | 0.85 (0.69-1.04) | 0.12 |
| HFpEF | 0.79 (0.67-0.94) | 0.009 |
| **Cardiovascular death** | | |
| Age * | 2.55 (2.04-3.22) | <0.001 |
| Hypertension | 0.78 (0.63-0.97) | 0.025 |
| ACEI/ARB | 0.59 (0.49-0.72) | <0.001 |
| Anemia † | 1.63 (1.25-2.16) | <0.001 |
| Albumin ‡ | 1.47 (1.19-1.81) | <0.001 |
| BUN § | 1.80 (1.44-2.26) | <0.001 |
| eGFR ‖ | 1.42 (1.13-1.77) | 0.002 |
| LVEF category | | |
| HFrEF | reference | |
| HFmrEF | 0.75 (0.58-0.97) | 0.031 |
| HFpEF | 0.62 (0.50-0.77) | <0.001 |
| **Non-cardiovascular death** | | |
| Age * | 2.12 (1.61-2.83) | <0.001 |
| Men | 0.58 (0.45-0.75) | <0.001 |
| ACEI/ARB | 0.63 (0.49-0.81) | <0.001 |
| Anemia † | 1.85 (1.30-2.71) | <0.001 |
| Albumin ‡ | 2.38 (1.85-3.05) | <0.001 |
| BUN § | 1.35 (1.03-1.78) | 0.031 |
| LVEF category | | |

© 2020 Kitai T et al. *JAMA Network Open.*

| | | |
|---|---|---|
| HFrEF | reference | |
| HFmrEF | 1.06 (0.74-1.51) | 0.75 |
| HFpEF | 1.19 (0.89-1.59) | 0.25 |

A total of 3,202 patients' data was available in this analysis.

* Age >80 years; † Defined by the World Health Organization criteria (hemoglobin <12 g/dL for women and <13 g/dL for men); ‡ Serum albumin level <3.0 g/dL; § BUN >25 mg/dL; || eGFR <30 mL/min/1.73m$^2$. HFrEF=heart failure with reduced ejection fraction; HFmrEF= heart failure with mid-range ejection fraction; HFpEF= heart failure with preserved ejection fraction; HR = hazard ratio; CI = confidence interval; ACEI = angiotensin converting enzyme inhibitor; ARB = angiotensin receptor blocker; MRA = mineralocorticoid receptor antagonist; BUN = blood urea nitrogen; and eGFR = estimated glomerular filtration

© 2020 Kitai T et al. *JAMA Network Open.*

**eTable 2.** Multivariable Analyses for Predicting Each Mode of Death in Patients With HFrEF

| | adjusted HR (95%CI) | P value |
|---|---|---|
| **All-cause death** | | |
| Age* | 2.85 (2.15-3.81) | <0.001 |
| Beta blocker | 0.70 (0.53-0.92) | 0.011 |
| ACEI/ARB | 0.58 (0.45-0.75) | <0.001 |
| Anemia † | 1.90 (1.35-2.74) | <0.001 |
| Albumin ‡ | 1.51 (1.14-1.99) | 0.004 |
| BUN § | 1.63 (1.21-2.20) | 0.001 |
| **Cardiovascular death** | | |
| Age * | 2.70 (1.93-3.81) | <0.001 |
| Hypertension | 0.72 (0.82-0.99) | 0.045 |
| Beta blocker | 0.70 (0.53-0.92) | 0.011 |
| ACEI/ARB | 0.58 (0.45-0.75) | <0.001 |
| MRA | 0.70 (0.51-0.96) | 0.028 |
| Anemia † | 2.08 (1.36-3.26) | 0.001 |
| BUN § | 1.52 (1.06-2.20) | 0.007 |
| eGFR ‖ | 1.65 (1.15-2.37) | <0.001 |
| **Non-cardiovascular death** | | |
| Age * | 3.17 (1.89-5.44) | <0.001 |
| Atrial fibrillation | 1.75 (1.05-3.07) | 0.032 |
| ACEI/ARB | 0.60 (0.38-0.95) | 0.031 |
| Albumin ‡ | 2.67 (1.66-4.25) | <0.001 |
| BUN § | 1.88 (1.13-3.13) | 0.014 |

A total of 1,177 patients' data was available in this analysis.

* Age >80 years; † Defined by the World Health Organization criteria (hemoglobin <12 g/dL for women and <13 g/dL for men); ‡ Serum albumin level <3.0 g/dL; § BUN >25 mg/dL; || eGFR <30 mL/min/1.73m$^2$. HFrEF=heart failure with reduced ejection fraction; HFmrEF= heart failure with mid-range ejection fraction; HFpEF= heart failure with preserved ejection fraction; HR = hazard ratio; CI = confidence interval; ACEI = angiotensin converting enzyme inhibitor; ARB **eTable 3.** Multivariable Analyses for Predicting Each Mode of Death in Patients With HFmrEF= angiotensin receptor blocker; MRA = mineralocorticoid receptor antagonist; BUN = blood urea nitrogen; and

© 2020 Kitai T et al. *JAMA Network Open.*

eGFR = estimated glomerular filtration

© 2020 Kitai T et al. *JAMA Network Open.*

**eTable 3.** Multivariable Analyses for Predicting Each Mode of Death in Patients With HFmrEF

| | adjusted HR (95%CI) | P value |
|---|---|---|
| **All-cause death** | | |
| Age* | 2.62 (1.75-4.00) | <0.001 |
| Beta blocker | 0.69 (0.49-0.99) | 0.045 |
| ACEI/ARB | 0.52 (0.36-0.74) | <0.001 |
| Albumin ‡ | 2.09 (1.45-2.99) | <0.001 |
| BUN § | 1.86 (1.24-2.82) | 0.003 |
| **Cardiovascular death** | | |
| Age * | 2.19 (1.33-3.74) | 0.002 |
| ACEI/ARB | 0.52 (0.33-0.82) | 0.004 |
| Albumin ‡ | 1.80 (1.11-2.85) | 0.018 |
| BUN § | 2.09 (1.25-3.57) | 0.005 |
| **Non-cardiovascular death** | | |
| Age * | 3.58 (1.85-7.29) | 0.001 |
| Men | 0.37 (0.19-0.68) | 0.001 |
| ACEI/ARB | 0.50 (0.27-0.89) | 0.019 |
| Albumin ‡ | 2.68 (1.50-4.75) | 0.001 |

A total of 618 patients' data was available in this analysis.

* Age >80 years; † Defined by the World Health Organization criteria (hemoglobin <12 g/dL for women and <13 g/dL for men); ‡ Serum albumin level <3.0 g/dL; § BUN >25 mg/dL; || eGFR <30 mL/min/1.73m$^2$. HFrEF=heart failure with reduced ejection fraction; HFmrEF= heart failure with mid-range ejection fraction; HFpEF= heart failure with preserved ejection fraction; HR = hazard ratio; CI = confidence interval; ACEI = angiotensin converting enzyme inhibitor; ARB = angiotensin receptor blocker; MRA = mineralocorticoid receptor antagonist; BUN = blood urea nitrogen; and eGFR = estimated glomerular filtration

© 2020 Kitai T et al. *JAMA Network Open.*

**eTable 4.** Multivariable Analyses for Predicting Each Mode of Death in Patients With HFpEF

| | adjusted HR (95%CI) | P value |
|---|---|---|
| **All-cause death** | | |
| Age* | 1.94 (1.49-2.54) | <0.001 |
| Men | 0.78 (0.63-0.97) | 0.028 |
| ACEI/ARB | 0.68 (0.54-0.85) | <0.001 |
| Anemia † | 1.67 (1.20-2.37) | 0.002 |
| Albumin ‡ | 1.87 (1.48-2.36) | <0.001 |
| BUN § | 1.53 (1.19-1.97) | 0.001 |
| eGFR ǁ | 1.41 (1.08-1.83) | 0.011 |
| **Cardiovascular death** | | |
| Age * | 2.56 (1.76-3.80) | <0.001 |
| Hypertension | 0.75 (0.61-0.93) | 0.008 |
| ACEI/ARB | 0.68 (0.54-0.85) | <0.001 |
| MRA | 0.64 (0.47-0.86) | 0.003 |
| Albumin ‡ | 1.68 (1.22-2.29) | 0.002 |
| BUN § | 2.01 (1.42-2.87) | <0.001 |
| eGFR ǁ | 1.50 (1.07-2.11) | 0.020 |
| **Non-cardiovascular death** | | |
| Men | 0.60 (0.43-0.84) | 0.003 |
| ACEI/ARB | 0.68 (0.54-0.85) | <0.001 |
| MRA | 0.64 (0.47-0.86) | 0.003 |
| Anemia † | 2.17 (1.28-3.93) | 0.003 |
| Albumin ‡ | 2.19 (1.54-3.09) | <0.001 |

A total of 1,407 patients' data was available in this analysis.

* Age >80 years; † Defined by the World Health Organization criteria (hemoglobin <12 g/dL for women and <13 g/dL for men); ‡ Serum albumin level <3.0 g/dL; § BUN >25 mg/dL; || eGFR <30 mL/min/1.73m$^2$. HFrEF=heart failure with reduced ejection fraction; HFmrEF= heart failure with mid-range ejection fraction; HFpEF= heart failure with preserved ejection fraction; HR = hazard ratio; CI = confidence interval; ACEI = angiotensin converting enzyme inhibitor; ARB = angiotensin receptor blocker; MRA = mineralocorticoid receptor antagonist; BUN = blood urea nitrogen; and eGFR = estimated glomerular filtration

© 2020 Kitai T et al. *JAMA Network Open.*
